# Histological changes of semitendinosus autograft after anterior cruciate ligament reconstruction in an immature rabbit model

**DOI:** 10.1186/s40634-015-0033-1

**Published:** 2015-08-28

**Authors:** Marco Giordano, Francesco Falciglia, Alessia Poggiaroni, Angelo Gabriele Aulisa, Pietro Savignoni, Vincenzo Guzzanti

**Affiliations:** Orthopaedics and Traumatology Division, Bambino Gesù Children’s Hospital, Institute of Scientific Research, Piazza San Onofrio 4, I-00165 Rome, Italy; University of Cassino (FR), Cassino, Italy

**Keywords:** Immature animal models, Anterior cruciate ligament, Ligamentization, Intra-articular tract, Semitendinosus tendon

## Abstract

**Introduction:**

The anterior cruciate ligament (ACL) injury is one of the most common in the knee. Tendons can be used as alternative grafts for ACL repair, with tendon “ligamentization” often reported in literature. The purpose of this study was to evaluate the morphological and histological changes occurring in a semitendinosus tendon (ST) during ACL reconstruction in growing rabbits.

**Materials and methods:**

Twenty-one 8-week-old New Zealand white rabbits, weighing about 1500 g underwent reconstructive surgery on the right knee. In two cases the left knee was used to verify the normal microstructure of the ACL and ST in rabbits. The rabbits were then randomly divided into seven groups and sacrificed at 1, 4, 6, 8, 12, 24 and 48 weeks after surgery. The specimens were evaluated under light microscopy to analyze the changes in the intra-articular tract of the graft. The evidence of necrosis, neovascularization and organization of the collagen fibers were investigated.

**Results:**

One month after surgery, numerous disorganized fibroblasts and collagenous fibers were identified. A marked reduction of cellular necrosis was observed in the early phase of the neo-ligament healing process. After 4 weeks, these fusiform-like cells became more rounded. By 8 weeks, the collagen fibers had become aligned in parallel with newly formed capillaries and highly differentiated fibroblasts. At 24 and 48 weeks the transplanted tendon differed histologically from both tendon and ligament.

**Conclusions:**

The data of the present study showed that ligamentization did not occur until at least 24 months post-operatively and, during healing, the grafted tendon assumed a unique micro-architecture that was a middle between a tendon and a ligament.

The ACL reconstruction in pediatric age has become more frequent in these past recent years. The use of semitendinosus graft with preservation of its distal attachment should be the gold standard in skeletally immature patients.

## Background

Anterior cruciate ligament (ACL) injuries in the skeletally immature athletes have been reported with increasing frequency, accounting for 31 % of the total knee injuries (Shea et al. 2004). ACL reconstruction in adolescents is still discussed. Non operative management is an appealing option in reason of the risk of physeal damage, however the recent literature uniformly indicates that conservative treatment of ACL tears in children results in a higher rate of instability that may progress to intra-articular damage, including meniscal tears (Fabricant et al. 2013; Henry et al. 2009; Ramsky et al. 2013). For this reason, there has been an increasing interest in the early operative management to restore stability in skeletally immature knees (Falciglia et al. 2014). Several aspects of the biology and biomechanics of the reconstruction of the anterior cruciate ligament have been underlined, but only few studies have been conducted on the process of ligamentization in childhood.

Although tendons have different both biological and histological features compared to ligaments, numerous studies have reported the use of tendons for ligamentous repair.

One of the still debatable questions is to clarify how the so-called “neo-ligament” really progressively loses its tendon specific biological characteristics exhibiting ligamentous histological properties. While there has been a wide number of studies exploring the various aspects of ACL reconstruction in adults only limited data are available regarding the human or animal tendon graft healing process in skeletally immature patients.

In the early twentieth century Roux, Bertocchi-Bianchetti, and Vallone published the first experimental studies on the histological characteristics of tendon grafts (Amiel et al. 1986).

Later, Carnevali indicated that the graft preserved its original features on its intra-articular tract (Claes et al. 2011). Amiel (1986) proposed the concept of graft ligamentization.

In a recent review, Claes examined several animal studies and showed that the implanted tendons indeed seemed to remodel into a ligamentous ACL-like structure (Claes et al. 2011; Scheffler et al. 2008). ACL reconstruction in children and adolescents with open physes requires physeal sparing procedures. Surgery should respect the biology and biomechanics of reconstruction of the anterior cruciate ligament in order to minimize the surgeons fear regarding the possibility of consequences on the growth of the lower limbs.

Different tendons have been proposed for surgical ACL reconstruction in skeletally immature patients but the semitendinosus is certainly the most commonly used. The purpose of this study was to evaluate the morphological and histological changes that occur in the intra-articular tract of a semitendinosus tendon, when used to reconstruct the ACL in growing rabbits, preserving its distal insertion. The study was performed on growing animals to determine the type and the extent of the lesions that develop in the intrarticular tract. Immature animals should have a greater potential for tissue regeneration. The hypothesis was that the so-called graft ligamentization process never completes beyond what was already stated during the maturation phase in other experimental studies in mature animal models.

## Methods

This experimental study is an histological qualitative description of the ultrastructural changes of semitendinosus autograft used for the anterior cruciate ligament reconstruction in an immature rabbit model. The experiment was approved by the local ethics committee and followed the guidelines for the care and use of animals at our institution. The principles of laboratory animal care were followed, as well as specific national laws where applied.

All rabbits used for the study were provided by the animal enclosure of the Catholic University of the Sacred Heart of Rome (Rome, Italy). The animals received antibiotic prophylaxis with 10 mg/kg amoxicillin (Amoxysol - Bayer Spa) (preoperative administration and two additional doses at intervals of 72 h) and analgesic therapy with 30 mg ketorolac (Toradol - Recordati Spa) for 2 days postoperatively. All the healthy rabbits were enrolled in the study. Exclusion criteria were limping, pain or other signs of disease as septic arthritis.

They have been observed for 1 week following surgery in adequate cages and then sent back to the farm under veterinarian care.

**Fig. 1:**
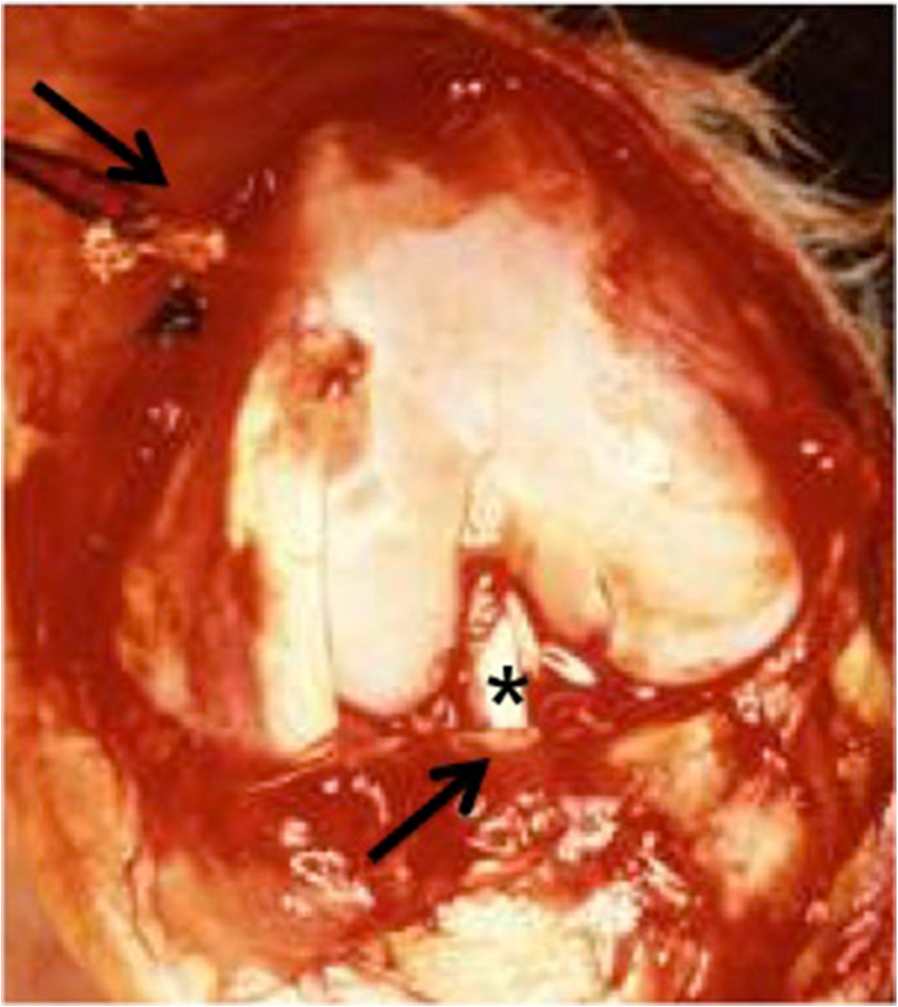
Carnevali’s technique: intraoperative field. The right knees joint was exposed. The ST and G tendons are divided from the musculo-tendinous junction, preserving the distal insertion site. Once the ACL was accurately removed the oblique 2-mm-diameter tunnels were drilled, mediolaterally in the tibia and anteroposteriorly in the femur (arrows), passing through the original attachments of the ACL and through the growth plates. Then, the semitendinosus tendon (*) was threaded through the tibial and femoral tunnels and then sutured to the periosteum of the lateral part of the femoral metaphysis with 2.0 silk thread

In 1934 Galeazzi proposed the anterior cruciate ligament reconstruction in humans using the semitendinosus tendon (Galeazzi 1934). Carnevali used this technique in animal models in 1938 (Carnevali 1938). For the study twenty-one 8-week-old New Zeland white rabbits, weighing about 1500 ± 200 g, underwent surgery on the right knee using this last technique (Fig. 1). The left knee was used to compare. The animals were anaesthetized with diazepam (Ziapam – Laboratoire Tvm) (3 to 5 mg/kg) and with intramuscular ketamine (Inoketam – Virbac Srl) (500 mg/kg).

The right knees joints were exposed through a medial parapatellar incision. The ACL was accurately removed and the original footprints were marked using a sterile pencil. Oblique 2-mm-diameter tunnels were drilled, mediolaterally in the tibia and anteroposteriorly in the femur, passing through the original attachments of the ligament and through the growth plates. Then, the semitendinosus tendon was cut at its proximal muscle-tendon junction, but left uninjured at its distal attachment site. The tendon was then threaded through the tibial and femoral tunnels and then sutured to the periosteum of the lateral part of the femoral metaphysis with 2.0 silk thread. The operated knees were immobilized in a cast for seven days, with all efforts made to minimize suffering and to avoid weight bearing. After surgery, the animals returned to their cages and were feeded with water and standard rabbit diet “ad libitum”.

**Fig. 2:**
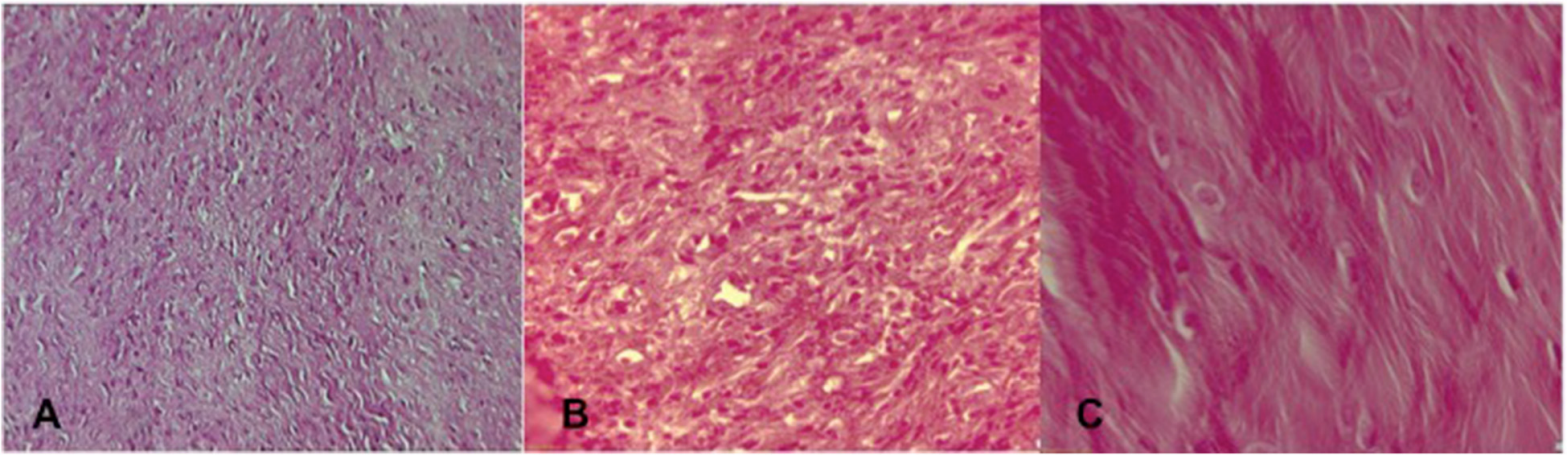
Normal structure of the anterior cruciate ligament in the growing rabbit. H&E staining (**a**) Original magnification x20; (**b**) Original magnification x60; (**c**) Original magnification x100

The animals were then randomly divided into seven groups of three and sacrificed at 1, 4, 6, 8, 12, 24 and 48 weeks after surgery. The right limb of each animal was dissected, measured with a caliper (DIN 862, Mauser-Messzeug-GMBH, Oberndorf/Neckar, Germany) and radiographed to detect deformity and leg-length discrepancy. In two cases the left knee was used to compare the normal ultrastructure of the ACL in rabbits (Fig. 2).

For the experimental and control limbs, the samples were fixed in 10 % formalin. Later they were decalcified in EDTA and embedded in paraffin. Longitudinal 5 μm-thick sections were stained with hematoxylin and eosin (H&E), Masson’s trichrome, Alcian-PAS and Alcian Blue with different MgCl_2_ concentrations (Quintarelli and Dellovo 1965). The specimens were evaluated under light microscopy (Leica DM RB) to analyze changes in the intra-articular tract of the graft. All specimens were compared with the normal rabbit ACL structure.

### Statistic

Quantitative variables were presented as means and SD or proportion where appropriate. The variables that the study had taken in account and qualitatively described were the following: necrosis, neovascularization and organization of the collagen fibers.

## Results

**Fig. 3:**
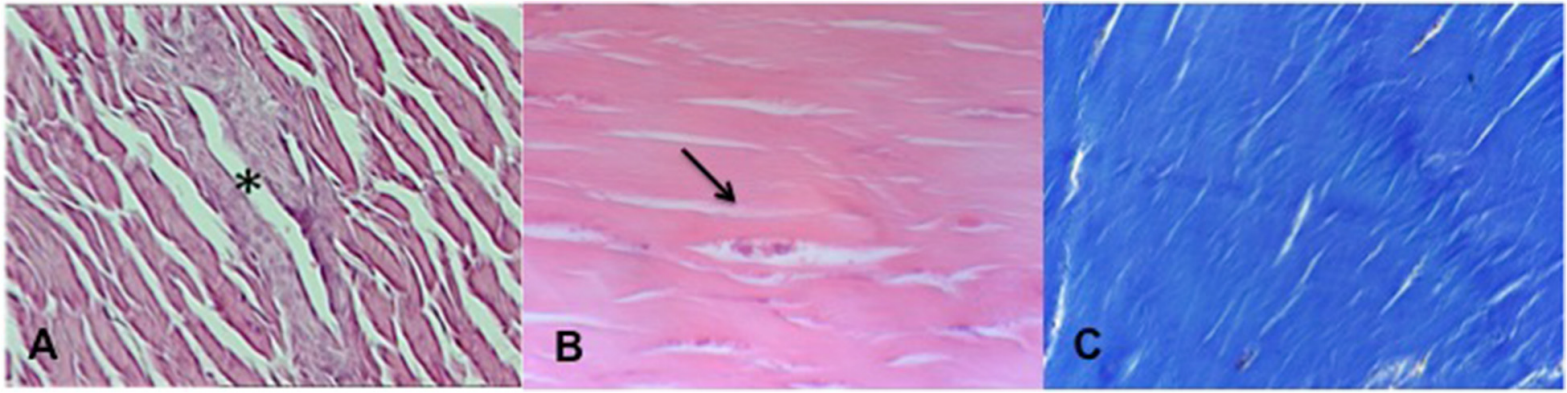
H&E staining (**a**) (Original magnification x40) One month after surgery the graft tissue showed regions of cell-free areas and necrosis. *Vessel located in the periphery of the graft and runned parallel to the collagen fiber bundles in the septa at forty days; **b** (Original magnification x100) fusiform fibroblast (arrow) in the central portion of the tendon. **c** Masson trichrome staining (Original magnification x40) Disorganized collagenous fibers at 4 weeks

Any complication with the animals, throughout the entire period of the study, was observed. All grafts were intact at the time of evaluation and they did not have the pearly appearance of the normal ACL. No failure of the semitendinosus graft was observed at the sacrificed time. At 1 week after surgery, the graft tissue showed focal cell-free areas with poor necrosis. Tissue necrosis was better evaluable at 3–4 weeks, as the intense inflammatory response, but it was no longer detectable after 40 days from surgery. 1 month after surgery, many fibroblasts were present, and numerous disorganized collagenous fibers were observed. The fibrillar collagen, such as type I and III, are the essential building blocks that provide tendon and ligaments with their elasticity and strength. The fibroblastic cells had a central position in the tendon with an initial longitudinal arrangement. The vascularization was still poor and vessels were located on the grafts periphery (Fig. 3a).

In the semitendinosus tendon, the fibroblastic cells appeared fusiform (Fig. 3b), which was different from the ACL rounder fibroblasts. However, after 4 weeks, these fusiform-like cells became more rounded. The collagen fibers became more organized after the first month with areas of disorganized collagen matrix (Fig. 3c). From the fourth to the eighth week after surgery the typical inflammatory cell population gradually disappeared and the small necrosis areas were not detectable.

**Fig. 4:**
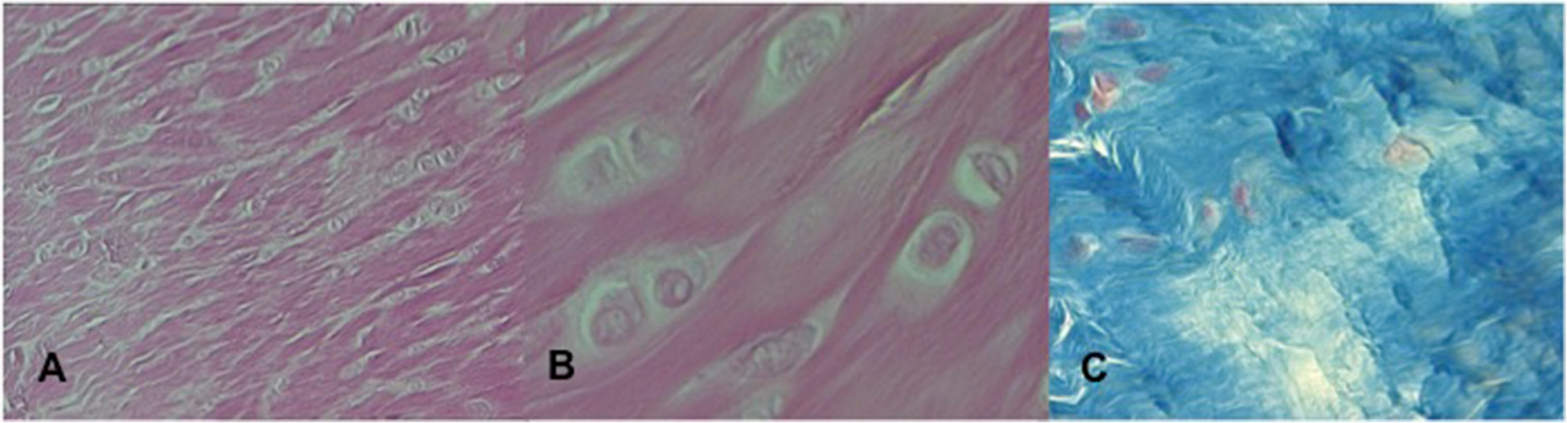
H&E staining (**a**) (Original magnification x40). Fibroblastic cells of the semitendinosus tendon at 2 months after surgery. **b** (Original magnification x100). Fibroblastic cells with large nuclei and abundant cytoplasm 8 weeks after surgery. **c** Masson trichrome staining (Original magnification x60) Collagenous fibers of the semitendinosus tendon at 8 weeks after surgery

**Fig. 5:**
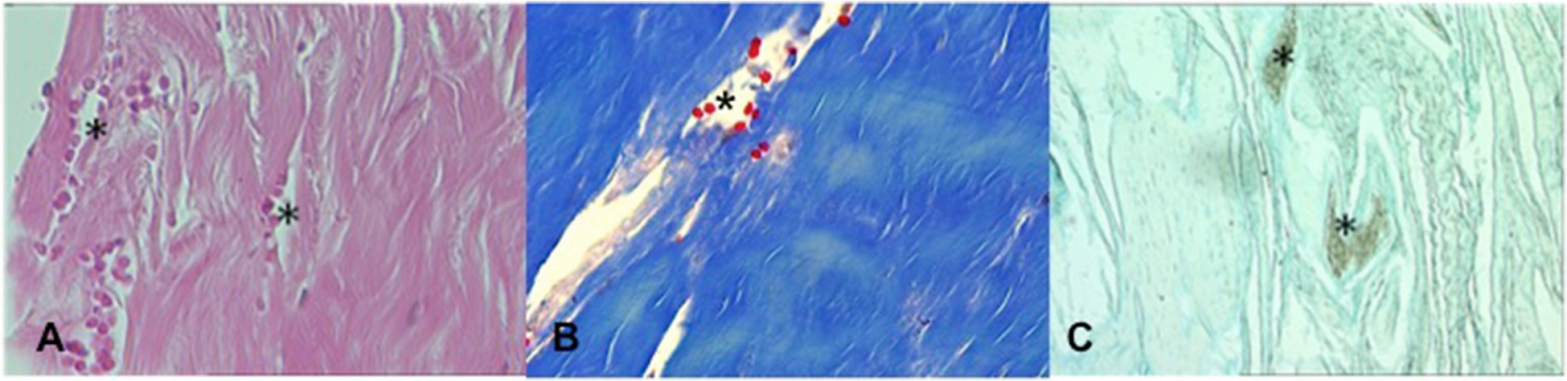
Peripheral and core vessels(*) in the semitendinosus tendon 24 weeks after surgery. **a** H&E staining (Original magnification x40). **b** Masson trichrome staining (Original magnification x40) (**c**) Alcian Blue (Original magnification x20)

**Fig. 6:**
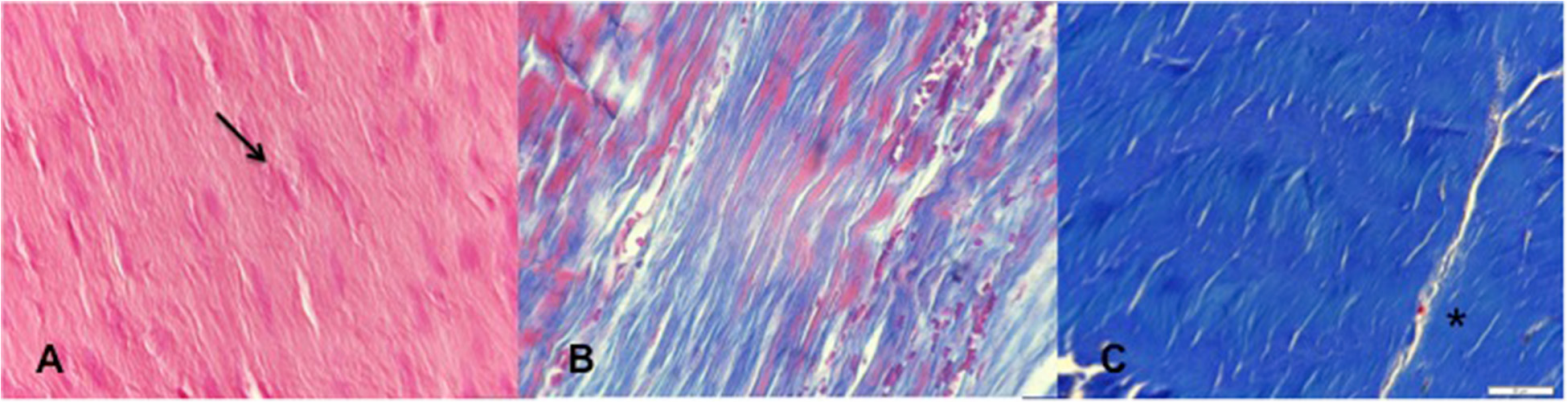
**a** H&E staining (Original magnification x60). Despite the histological changes the neoligament at 48 weeks after surgery does not assume the characteristics of the normal rabbits ACL. The fibers showed a slight separation with increased waviness. The cellularity and vascularity were graded as normal. The fibrocytes have different characteristics from the fibroblastic cells observed few time after surgery. **b** Masson trichrome staining (Original magnification x20). Semitendinosus tendon 48 weeks after surgery. An increased longitudinal and parallel orientation of the collagen bundles was observed; **c** Masson trichrome staining (Original magnification x40). At 48 weeks the neoligament was viable. Red cells were present in the peripheric portion of the graft

At 8 weeks after surgery, the collagen fibers aligned in parallel with the new capillaries and with the fibroblastic cells that had become highly differentiated (Fig. 4). The semitendinosus tendon, which initially had no vessels after grafting, gradually became vascularized, expecially after 40 days after surgery. The grafts, however, did not appear to resemble the ACL at this timepoint, with the mean graft cross-sectional area significantly higher in the operated knee as compared with the normal left knee ligament. At the end of the healing process of the neo-ligament (24 weeks) the loss of the regular collagen orientation and crimp pattern was observed with the shift of the diameter of the collagen fibrils, from large to small, the reduction of collagen type I fibrils and an increase of collagen type III synthesis. The histological features observed in the sacrificed animals at 24 weeks (Fig. 5) were not subject to further changes at 48 weeks (Fig. 6). Between 24 and 48 weeks the graft had not yet acquired the characteristics of the normal rabbit ACL in the intra-articular tract of the sections. Table 1 summarizes the major histological characteristics at each time-point.

**Table 1:** Major histological characteristics at each time-point in ST graft

	1 week	4 weeks	6 weeks	8 weeks	12 weeks	24 weeks	48 weeks
Necrosis	Focal cell-free areas with small necrosis	Cell-free areas with poor necrosis	Gradually disappeares		No necrosis sign		
Neovascularization	No vessel	Poor and located on the grafts periphery	Vessel located in the periphery of the graft and runned parallel to the collagen fiber bundles in the septa	Vessel located from periphery to central area	Normal vascularity		
Organization of the collagen fibers	ST tendon characteristics	Disorganized collagenous fibers	Initial longitudinal arrangement	Collagen fibers aligned in parallel with the new capillaries and with the fibroblastic cells	Loss of regular collagen orientation and crimp pattern	Slight separation with increased waviness of fibers	
					Shift of the diameter of the collagen fibrils, from large to small	Unimodal appearance of collagen fibrils	
					Reduction of collagen type I fibrils	Failure restauration of collagen orientation to normality	
					Increase of collagen type III synthesis		
Cellularity	Inflammatory cell population		Aligned longitudinally, more rounded fibroblastic cells with large nuclei and abundant cytoplasm	Highly differentiated fibroblastic cells	Normal cellularity		
	Disorganized and randomly arranged fusiform-like fibroblastic cells with plump nuclei			Gradual disappearance of the typical inflammatory cell population	Fibrocyte have different characteristics from fibroblastic cells		

## Discussion

The most noteworthy findings in the current study were: 1) The semitendinosus graft did not undergo full ligamentization and, after 48 weeks, its original appearance at the microstructural level was different; 2) A marked reduction of cellular necrosis was observed in the early phase of the neo-ligament healing process.

The ACL reconstruction in pediatric age has become more frequent in these past recent years. In skeletally immature patients, physeal-sparing procedures are recommended for the reconstruction of the injured ACL. The use of patellar tendon should be avoided for the potential risk of premature physeal closure and subsequent tibial recurvatum (Meller et al. 2008), moreover it may interfere with the delicate changes of the extensor mechanism during growth (progressive reduction of laxity, reduction of Q angle, lower limb torsional changes and gradual patella lowering). For these reasons the semitendinous graft is widely used for ACL reconstruction in children and teenage. Carnevali’s surgical technique, used in this study, satisfies these requirements.

The ligamentization process was never evaluated on skeletally immature rabbits before. In Literature there were only a few papers on growing animal models, but no studies on growing rabbits has ever been performed before. This last aspect makes the study on skeletally immature patients original. In particular in other studies, the experiments were performed on mature rabbits (Amiel et al 1986; Blickenstaff et al. 1997; Papachristou et al. 2007; Tohyama et al. 2006); in others immature sheep (Meller et al. 2009), mature sheep (Blickenstaff et al. 1997; Mayr et al. 2012) or an immature canine model (Chudik et al. 2007). Besides, while we used the semitendinosus tendon, other studies used the Achilleous tendon (Li et al. 2009) the patellar tendon (Amiel et al. 1986; Falconiero et al. 1998), the superficial flexor digitorum tendon and the gastrocnemius tendon (Li et al. 2009). Few studies considered the remodeling of autografts after anterior cruciate ligament reconstruction in mature human patients (Claes et al. 2011; Scheffler et al. 2008).

Amiel (1986) proposed the concept of graft ligamentization. In his study, he used a rabbit model for ACL reconstruction and transplanted the autogenous patellar tendon in 37 adult rabbits of approximately 6 to 8 months old. He showed that patellar autograft underwent a process of “ligamentization” when placed in the ACL environment and that the cells responsible for this change were of an extragraft origin. He concluded that the ligamentization process was a continuous development of the tendon into a substance similar to a normal ligament. Other studies supported these findings (Falconiero et al. 1998; Marumo et al. 2005; Sanchez et al. 2010).

On the opposite side, Bosch demonstrated that the autograft tissue was different from the original tendon both structurally and mechanically, and that it would never approach the characteristics of a normal ligament (Bosch and Kasperczyk 1992). He also stressed the importance of the four different phases of the autograft healing process: necrosis, revitalization, collagen formation and remodeling. This was supported by Blickenstaff, who concluded that, at the end of the healing period of 52 weeks, the graft was different from the original ligament, but also had different characteristics from the original tendon (Blickenstaff et al. 1997).

Kennedy analyzed the evolution of intra-articular semitendinosus (ST) transfer in rabbits concluding that the surgical ACL reconstruction was a failure (Kennedy et al. 1980). In accordance with Amiel’s paper, Tohyama published a study showing that the patellar tendon graft underwent important changes in its structure that could be divided into the upper mentioned four stages (Tohyama et al. 2006). He concluded that the matrix changes were caused by the adaptation of the patellar tendon to the ACL environment.

In literature only one experimental study on rabbit models used the semitendinosus tendon for ACL reconstruction with preservation of the distal insertion. In this study the specimens were taken up to 12 weeks after surgery, instead in our study they were extended beyond 24 weeks after surgery reaching the rabbits skeletal maturity, which verifies approximately at about 28 weeks. (Masoud et al. 1986 and Kaweblum et al. 1994). Papachristou immobilized rabbits for 2 weeks after surgery, instead in our study we immobilized the animals for only one week to avoid the potential risk of osteonecrosis. Despite the time points of the samples explanted were not completely overlapped, in the first month after surgery the results were similar (absence of avascular necrosis, disorganized collagen fibers). Papachristou described average or intense vascularization with new formed vessels at 3 weeks. This last finding, in our study, was observed between the 4^th^ and the 6^th^ week. The phase of cell differentiation was more markedly observed between the 6^th^ and the 12^th^ week. After this time point we evidenced the maturation phase of the graft which maintained vital up to 48 weeks after surgery.

Our study analyzed the trend and the graft patterns across different timepoints to verify the microscopic changes that occurred during the healing process. After surgery, the space between the graft and the original bone was filled with a fibrovascular tissue. The healing process occurred by the establishment of collagen fibers and neovascularization. The graft showed a fibroblasts number increase during the early postoperative period (about 1 month). The fibroblastic cells appeared disorganized and randomly arranged (Fig. 3). It was believed that the cells were metabolically active because of the presence of plump nuclei. After this timepoint, the fibroblasts aligned longitudinally became rounded and their nuclei less ovoidal (Fig. 4).

The study confirmed, even in growing rabbits, that the findings reported by other authors such as the graft vascularization began only 30–40 days after surgery (Amiel et al. 1986) and the initial focal necrosis areas were no more detectable after 4–6 weeks (Ekdahl et al. 2008; Stener et al. 2012). The new vessels initially appeared on the grafts periphery and later were present within the central zone (Fig. 4). Collagen orientation did not return to normality during the study period (48 weeks). Moreover, at the electron microscopic analysis, the diameter of the collagen fibrils lost their normal bimodality appearance changing in unimodal as described by Abe et al. (1993).

As a collateral finding, although it was not the aim of this study, any necrosis sign in the physis was observed. Therefore, drilling a tunnel through the region of the growth plate did not alter bone growth; although, there was transient hypertrophy in the portion, which disappeared within the first month after surgery (Guzzanti et al. 1994).

The remodeling phase of the healing process of the tendon graft is the most vulnerable phase because of the increased necrosis, revascularization and extra-cellular infiltration (Claes et al. 2011; Papachristou et al. 2007; Pauzenberger et al. 2013). Other animal studies have shown that the patellar tendon, similar to the semitendinosus tendon, underwent more extended avascular necrosis and revascularization after transplantation (Amiel et al. 1986; Bosch and Kasperczyk 1992). These histological observations, compared with human studies, showed that especially in the early phase there was a marked necrosis in the central portion of the graft (Amiel et al. 1986; Bosch and Kasperczyk 1992). Although the preservation of the distal insertion of the semitendinosus tendon did not improve the final graft histological feature, and even the time of the different phases of the remodeling process were not modified, however, this study noted a marked reduction in cellular necrosis, only in focal areas observed. This aspect was due to the increased vascularization of the neoligament directly given by the preservation of the distal attachment of the semitendinosus that improved both revascularization and growth of fibroblasts, as observed by Papachristou. This finding is very important because it reduces the vulnerability of the graft especially during the first weeks after ACL reconstruction.

Even though this study showed a lower percentage of cellular necrosis, it is better to be more cautious and avoid an early and intensive rehabilitation treatment in adolescents, this not for the histological weakness but for the uncertainty on their prudent post-surgical conduct.

A thorough knowledge of the graft healing process is necessary to improve aspects of the surgical technique and in particular for the rehabilitation program.

The understanding of the ligamentization process is still far from definitive answers. However one thing is clear: the neoligament doesn’t assume the histological characteristics of the normal anterior cruciate ligament, expressing always an intermediate structure between the tendon and the ligament. However, clinical experience in the use of the semitendinosus for the ACL injured reconstruction shows that the ultrastructural appearance of the graft, viable at any given time point of the studies, at the end of the healing process provides sufficient guarantees such as to justify its use.

The major limitation of our study was the lack of biomechanical analysis and the ability to transfer the findings of this animal model to human tendon transplantation surgeries. The core biopsy of the graft would be appropriate even in childhood. However, the minor surgical cases and the rarity of degenerative disease in this population make second-look arthroscopy extremely rare and conditioned by small percentage of biopsies taken from the center of the graft.

Findings showed in experimental animal models, in their general concept about the progressive biologic changes that develop in the transplanted semitendinosus tendon, could be useful to lead ACL reconstruction in pediatric and adolescent population.

## Conclusion

The ligamentization process is not present up to 48 weeks after surgery in growing rabbit models. The grafted ST tendon changes into a new entity during the healing process, assuming a micro-architecture somehow between the tendon and the ligament ones. Semitendinosus graft undergoes a process of adaptation rather than full restoration of the intact ACL’s biology properties.

The use of semitendinosus for ACL reconstruction with preservation of its distal insertion should be the gold standard in skeletally immature patients.

## Abbreviations

ACL: anterior cruciate ligament
ST: semitendinosus tendon
H&E: hematoxylin and eosin
